# Novel genetic risk variants for pediatric celiac disease

**DOI:** 10.1186/s40246-016-0091-1

**Published:** 2016-10-24

**Authors:** Angeliki Balasopoulou, Biljana Stanković, Angeliki Panagiotara, Gordana Nikčevic, Brock A. Peters, Anne John, Effrosyni Mendrinou, Apostolos Stratopoulos, Aigli Ioanna Legaki, Vasiliki Stathakopoulou, Aristoniki Tsolia, Nikolaos Govaris, Sofia Govari, Zoi Zagoriti, Konstantinos Poulas, Maria Kanariou, Nikki Constantinidou, Maro Krini, Kleopatra Spanou, Nedeljko Radlovic, Bassam R. Ali, Joseph Borg, Radoje Drmanac, George Chrousos, Sonja Pavlovic, Eleftheria Roma, Branka Zukic, George P. Patrinos, Theodora Katsila

**Affiliations:** 1 Department of Pharmacy, School of Health Sciences, University of Patras, University Campus, Rion, 265 04 Patras, Greece; 2 Institute of Molecular Genetics and Genetic Engineering, University of Belgrade, Belgrade, Serbia; 3 Complete Genomics Inc., Mountain View, CA USA; 4 BGI Shenzhen, Shenzhen, 51803 China; 5 Department of Pathology, College of Medicine and Health Sciences, United Arab Emirates University, Al Ain, United Arab Emirates; 6 Department of Immunology and Histocompatibility, “Aghia Sophia” Children’s Hospital, Athens, Greece; 7 First Department of Pediatrics, National and Kapodistrian University of Athens Medical School, Athens, Greece; 8 Department of Gastroenterology and Nutrition, University Children’s Hospital, Medical Faculty, University of Belgrade, Belgrade, Serbia; 9 Department of Applied Biomedical Science, Faculty of Health Sciences, University of Malta, Msida, Malta

**Keywords:** Celiac disease, Genomic variants, Family genomics, Next-generation sequencing, Disease predisposition

## Abstract

**Background:**

Celiac disease is a complex chronic immune-mediated disorder of the small intestine. Today, the pathobiology of the disease is unclear, perplexing differential diagnosis, patient stratification, and decision-making in the clinic.

**Methods:**

Herein, we adopted a next-generation sequencing approach in a celiac disease trio of Greek descent to identify all genomic variants with the potential of celiac disease predisposition.

**Results:**

Analysis revealed six genomic variants of prime interest: *SLC9A4* c.1919G>A, *KIAA1109* c.2933T>C and c.4268_4269delCCinsTA, *HoxB6* c.668C>A, *HoxD12* c.418G>A, and *NCK2* c.745_746delAAinsG, from which *NCK2* c.745_746delAAinsG is novel. Data validation in pediatric celiac disease patients of Greek (*n* = 109) and Serbian (*n* = 73) descent and their healthy counterparts (*n* = 111 and *n* = 32, respectively) indicated that *HoxD12* c.418G>A is more prevalent in celiac disease patients in the Serbian population (*P* < 0.01), while *NCK2* c.745_746delAAinsG is less prevalent in celiac disease patients rather than healthy individuals of Greek descent (*P* = 0.03). *SLC9A4* c.1919G>A and *KIAA1109* c.2933T>C and c.4268_4269delCCinsTA were more abundant in patients; nevertheless, they failed to show statistical significance.

**Conclusions:**

The next-generation sequencing-based family genomics approach described herein may serve as a paradigm towards the identification of novel functional variants with the aim of understanding complex disease pathobiology.

**Electronic supplementary material:**

The online version of this article (doi:10.1186/s40246-016-0091-1) contains supplementary material, which is available to authorized users.

## Background

Celiac disease is a complex chronic immune-mediated disorder of the small intestine. Today, the pathobiology of the disease is unclear, perplexing differential diagnosis, patient stratification, and decision-making in the clinic. Genetics has been reported to play a key role. The *HLA-DQ2* gene is identified in up to 95 % of celiac disease patients, while most of the remaining patients have the *HLA-DQ8* gene. Notwithstanding, the Chinese and Japanese populations (devoid of *HLA-DQ2*) are not expected to develop the disease, yet this is not true for the individuals with the *HLA-DQ8* gene. Celiac disease is also associated with an extended ancestral haplotype that is defined by class I and II *HLA*s (*A*, *B*, *DR*, *DQ*). Notably, *HLA-DQ2* and/or *HLA-DQ8* expression is necessary but not sufficient for disease development. Thus, other genes are anticipated to be involved. Indeed, genome-wide association studies (GWAS) have revealed 26 non-*HLA* genetic loci-associated celiac disease and other autoimmune or chronic immune disorders (diabetes mellitus type I, rheumatoid arthritis) [1, 2]. In 2008 to 2011, several new celiac disease risk loci have been identified [3–5], bringing the number of known loci (including the *HLA* one) to 40 and indicating genes and gene regulatory elements of paramount importance. In 2015, five new genetic loci were identified, being independent of *HLA-DQA1* and *HLA-DQB1* and associated with celiac disease predisposition [6].

Although a genetic component has been described, disease occurrence has been also associated with environmental factors and gut microbiome. In all cases, gluten has been identified as the environmental trigger of the disease, leading to the stimulation of gluten-specific T cells. Differential diagnosis is still a major issue. Although a gold standard diagnostic approach has been defined (endoscopy with biopsy of the small intestine coupled to positive disease serology), several pathological conditions have been reported sharing similar mucosal transformations with celiac disease as well as other autoimmune disorders (thyroid disease, Addison disease, autoimmune liver disease, Sjögren syndrome) that occur ten times more frequently in celiac disease patients often masking celiac disease symptoms. Disease management options are restricted to a gluten-free lifestyle, which ultimately fails to protect patients from disease symptoms due to its chronic nature. Can we delineate individual variability towards differential diagnosis? Can we highlight the disease mechanisms in question to assist disease management?

So far, findings account for 49 % of the genetic basis of the disease. As in other immune-mediated diseases, genetic predisposition to celiac disease remains unresolved as we still need to explain the remaining major fraction of heritability, including rare as well as additional common risk variants. Causal variants and genes still need to be identified and/or more finely localized. In this context, the Immunochip Consortium was developed to explore comprehensive datasets containing common, low-frequency, and rare variants in related diseases (autoimmune thyroid disease, ankylosing spondylitis, Crohn disease, celiac disease, IgA deficiency, multiple sclerosis, primary biliary cirrhosis, psoriasis, rheumatoid arthritis, systemic lupus erythematosus, type 1 diabetes mellitus, and ulcerative colitis) [7].

As expected, the advent of technology and, in particular, next-generation sequencing has provided unprecedented opportunities to delineate disease pathobiology as well as inter-individual differences [8, 9]. Herein, we propose a multi-step next-generation sequencing-based family genomics approach, piloted in a celiac disease trio of Greek descent to identify novel genomic variants of functional significance with the aim of understanding disease pathobiology.

## Methods

### Case selection, DNA isolation, and whole-genome sequencing

A seven-member Greek family has been recruited (informed consents have been obtained), and a trio analysis (III-1, III-2, IV-3) has been performed using the celiac disease model (Additional file 1: Figure S1). A family-based design was employed rather than a population-based design, as the former is generally considered to be robust against population admixture and stratification and may yield both within- and between-family information [10]. Genomic DNA isolation was performed from saliva using the Oragene collection kit (DNA Genotek, Ontario, Canada) (Serbian cohort) and peripheral blood using an automated system (MagNA Pure Compact, Roche, Basel, Switzerland) (Greek cohort). Whole-genome sequencing was performed using Complete Genomics’ (CA, USA) DNA nanoarray platform [11]. DNA sequencing coverage was 110×. Only high-quality call variants were included in the analysis (>93 %). Genomes were aligned with the hg19 reference genome.

### Bioinformatics and in silico analyses

Next-generation sequencing data (Complete Genomics Inc., CA) were analyzed using Ingenuity Variant Analysis version 3.1.2 (Ingenuity^®^ Systems, www.ingenuity.com). This is a well-established software that identifies associations between phenotypes, defined by the user by classification of the tested individuals, and variants in the sequenced genome. Upon classification of the family members by phenotype (celiac vs. normal), a number of variants were listed; the output was filtered into a smaller variant list upon classification of the family members by those being celiac patients vs. those who were healthy and known not to be celiac disease subjects. The genetic model used for this comparison was of an autosomal dominant model, since it traces the genetic inheritance from mother (III-2) to daughter (IV-3) in a highly penetrant form. A total of 263 genes followed an autosomal dominant pattern, and 227 variants were identified in the genetic model. Out of these, 6 genes and 7 variants were identified in the biological/phenotype pattern of celiac disease, due to either the past association of the genes considered or via the biological significance as determined by the IVA software.

Due to the large amount of variants which are normally present in the genome, even when excluding intronic sequences, several filtering steps are used in data analysis to include only the genes which are likely to be causative in the final output connected with just the disease of interest, in this case—celiac disease. All variants were filtered according to the analysis required, using custom scripts and Complete Genomics Analysis Tools (CGA™ Tools). The filtering cascade utilized in the present data analysis is as follows:

1. Confidence: Only variants with call quality at least 20.0 in cases or at least 20.0 in controls (i.e., 99 % call accuracy) were included.
2. Common variants: All variants observed to have an allele frequency ≥3.0 % of the genomes in the 1000 genomes project OR ≥3.0 % of the public Complete Genomics genomes OR ≥3.0 % of the NHLBI ESP exomes were excluded.
3. Predicted deleterious: Only variants that are experimentally observed to be associated with a phenotype (Pathogenic, Possibly Pathogenic, Unknown Significance OR established gain of function in the literature OR gene fusions OR inferred activating mutations by Ingenuity OR predicted gain of function by BSIFT OR in a microRNA binding site OR Frameshift, in-frame indel, or stop codon change OR Missense and not predicted to be innocuous by SIFT OR disrupt splice site up to 2.0 bases into intron OR deleterious to a microRNA OR structural variant) were kept.
4. Genetic analysis: Only variants with the following genotype characteristics were kept: associated with gain of function OR heterozygous_alt OR haploinsufficient OR heterozygous OR homozygous OR heterozygous_amb OR compound_heterozygous OR hemizygous AND occur in at least 2 of the case samples at the gene level in the Case samples AND not which are associated with gain of function OR heterozygous_alt OR haploinsufficient OR heterozygous OR homozygous OR heterozygous_amb OR compound_heterozygous OR hemizygous AND occur in at least 1 of the control samples at the variant level in the control samples.
5. Biological context: Only variants that are within 1 hop (direct targets) of upstream regulators or downstream regulatory targets of such genes and that are known or predicted to affect celiac disease.

Variants of interest were annotated with Annovar in Galaxy [12] and compared with NCBI dbSNP build 137 (http://www.ncbi.nlm.nih.gov/projects/SNP/snp_summary.cgi), 69 reference genomes from Complete Genomes (http://www.completegenomics.com/publicdata/69Genomes/), and GWAS (http://www.genome.gov/gwastudies) to determine their novelty or otherwise. To obtain a list of variants of potential functional significance, we employed protein variation effect analyzer (PROVEAN) v1.1.3 (PROVEAN human genome variants tool) that provides both scale-invariant feature transform (SIFT) [13] and PROVEAN [14] predictions for a given list of human genome variants as well as accessory information (dbSNP rs IDs, gene description, PFAM domain, GO terms, etc.). PROVEAN is able to make predictions for any type of protein sequence alteration, including single or multiple amino acid substitutions, deletions, and insertions [15]. Additionally, Variant Effect Predictor [16] and RegulomeDB [8] were employed to allow further data interrogation.

### Downstream molecular analysis

Selected variants were subsequently validated in pediatric celiac disease patients of Greek (*n* = 109) [17] and Serbian (*n* = 73) [18] descent and their healthy counterparts (*n* = 111 and *n* = 32, respectively). The diagnosis of celiac disease was based on the criteria of the European Society for Paediatric Gastroenterology, Hepatology and Nutrition (ESPGHAN) [19]. For children diagnosed prior to 1990, the “Interlaken criteria” were applied. The Ethics Committee of University Children’s Hospital, University of Belgrade, and the Review Board of “Aghia Sophia” Children’s Hospital have approved the study.

Amplification was carried out according to the KAPA2G Fast HotStart protocol (KAPABIOSYSTEMS, MA, USA); detailed information per SNP amplification conditions is available upon request. For *SLC9A4* c.1919G>A, an allele-specific polymerase chain reaction (PCR) assay was developed (two alternative reverse primers hybridizing exclusively either to the wild-type or the mutant allele). For *NCK2* c.745_746delAAinsG, PCR products were subjected to *XcmI* (New England Biolabs, MA, USA) restriction endonuclease analysis at 37 °C for 1.15 h and subsequent enzyme deactivation (65 °C, 20 min). Restriction fragments were visualized by 3 % agarose gel electrophoresis following ethidium bromide or Midori Green staining. For *HoxD12* c.418G>A, *HoxB6* c.668C>A, and *KIAA1109* c.2933T>C and c.4268_4269delCCinsTA, a PCR-based conventional Sanger resequencing approach was employed. Capillary electrophoresis was performed on the ABI Prism 3130xl DNA Analyzer (Applied Biosystems). Sanger sequencing was also employed to ensure PCR-RFLP and allele-specific PCR method verification.

### Statistical analysis

Herein, a thorough statistical review and analysis has been attempted, having always in mind that we used the celiac disease model (trio analysis) as a reason to conduct a more refined approach in searching/genotyping the resulted variants in a selective population of celiac disease of Greek and Serbian descent. We tested for deviations from HWE using the chi-square goodness of fit test and principal component analysis. Considering that the *χ* ^2^ approximation can be poor when there are low genotype counts, a Fisher exact test (R genetics package) was also used, as it does not rely on the *χ* ^2^ approximation [20]. Tests were performed as two-tailed, and differences were considered statistically significant when *P* < 0.05. Focusing on case-control phenotypes, we tested the null hypothesis of no association between rows and columns of the 2 × 3 matrix that contains the counts of the three genotypes (the two homozygotes and the heterozygote) among cases and controls. Again, a Fisher exact test was preferred (to evaluate genotype and allele frequencies), as it is computationally more demanding, but it is easily implemented in R. We also performed the Armitage test (Monte Carlo method; it obtains results that are closer to an exact test, since the classical Cochran-Armitage trend test is based on approximation) [20].

## Results and discussion

### Whole-genome sequencing analysis of trio reveals newly identified genetic risk variants for pediatric celiac disease

**Fig. 1:**
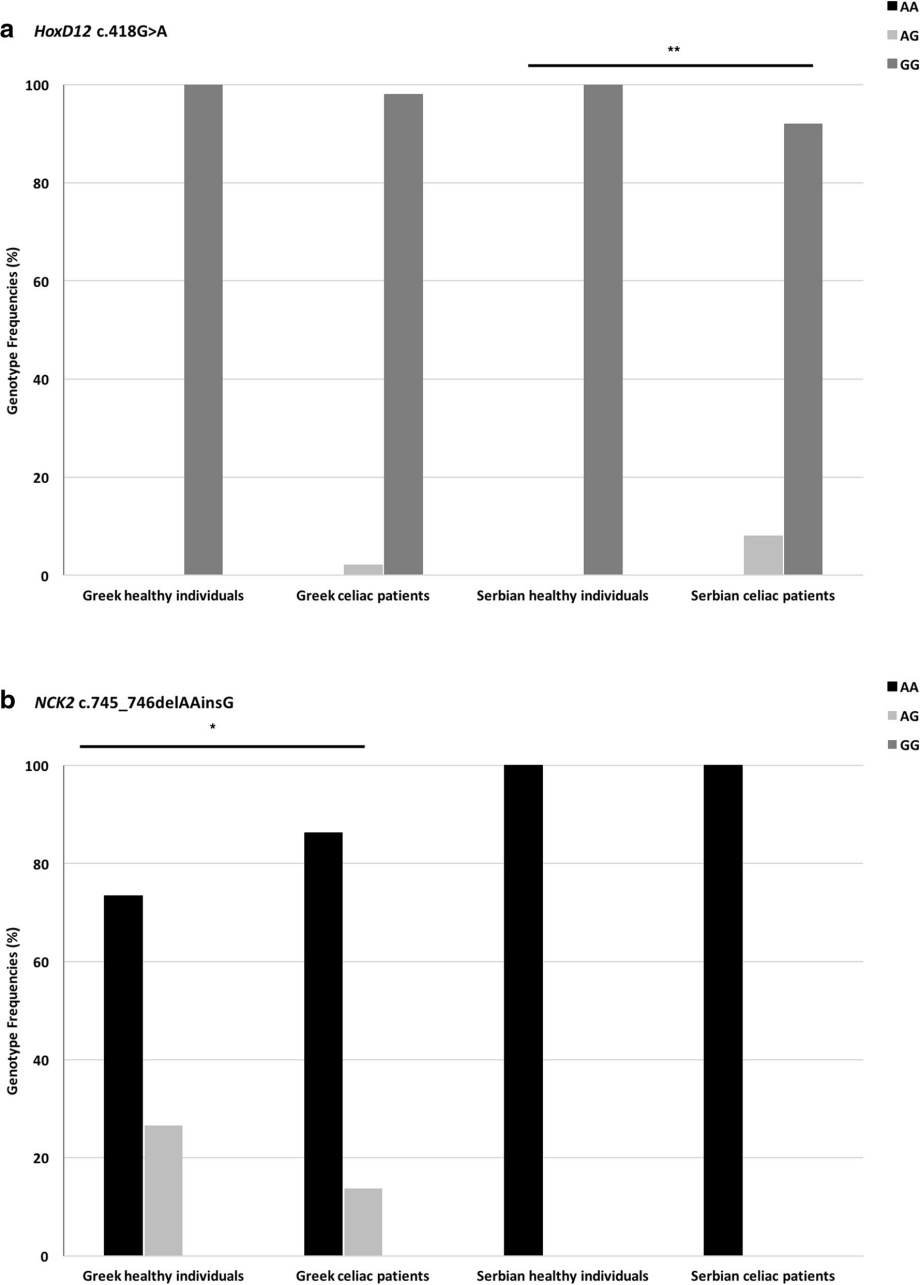
Distribution of newly identified risk variants in Greek and Serbian populations. **a** *HoxD12* c.418G>A is more abundant in pediatric celiac disease patients of Greek and Serbian descent, reaching statistical significance (**P < 0.01) in the Serbian population. **b** *NCK2* c.745_746delAAinsG is more abundant in healthy individuals of Greek and Serbian descent, reaching statistical significance (*P = 0.03) in the Greek population

Our multi-step next-generation sequencing-based family genomics approach revealed six genomic variants of prime interest: *SLC9A4* c.1919G>A, *HoxD12* c.418G>A, *KIAA1109* c.2933T>C and c.4268_4269delCCinsTA, *HoxB6* c.668C>A, and *NCK2* c.745_746delAAinsG. Susceptibility to celiac disease (CELIAC6) and to autoimmunity (AIS5) has been previously mapped to the 4q27 region, within a linkage disequilibrium block encompassing *KIAA1109*, *TENR*, *IL2*, and *IL21* genes [21]. So far, the most significant linkage outside the *HLA* region refers to rs13119723 (*P* = 2.0 × 10^−7^ in the *KIAA1109* gene on chromosome 4q27). Zhernakova and coworkers [2] hypothesized that the *KIAA1109*/*TENR*/*IL2*/*IL21* susceptibility region reported by van Heel and coworkers [21] might represent a general risk locus for multiple autoimmune diseases. Even though several CELIAC6 genomic variants have been reported [1, 2, 4, 21], this is the first time such a disease association is revealed for *KIAA1109* c.2933T>C and c.4268_4269delCCinsTA. *SLC9A4* c.1919G>A, *HoxD12* c.418G>A, *HoxB6* c.668C>A, and *NCK2* c.745_746delAAinsG are also reported for the first time as celiac disease risk variants (Fig. 1).

**Fig. 2:**
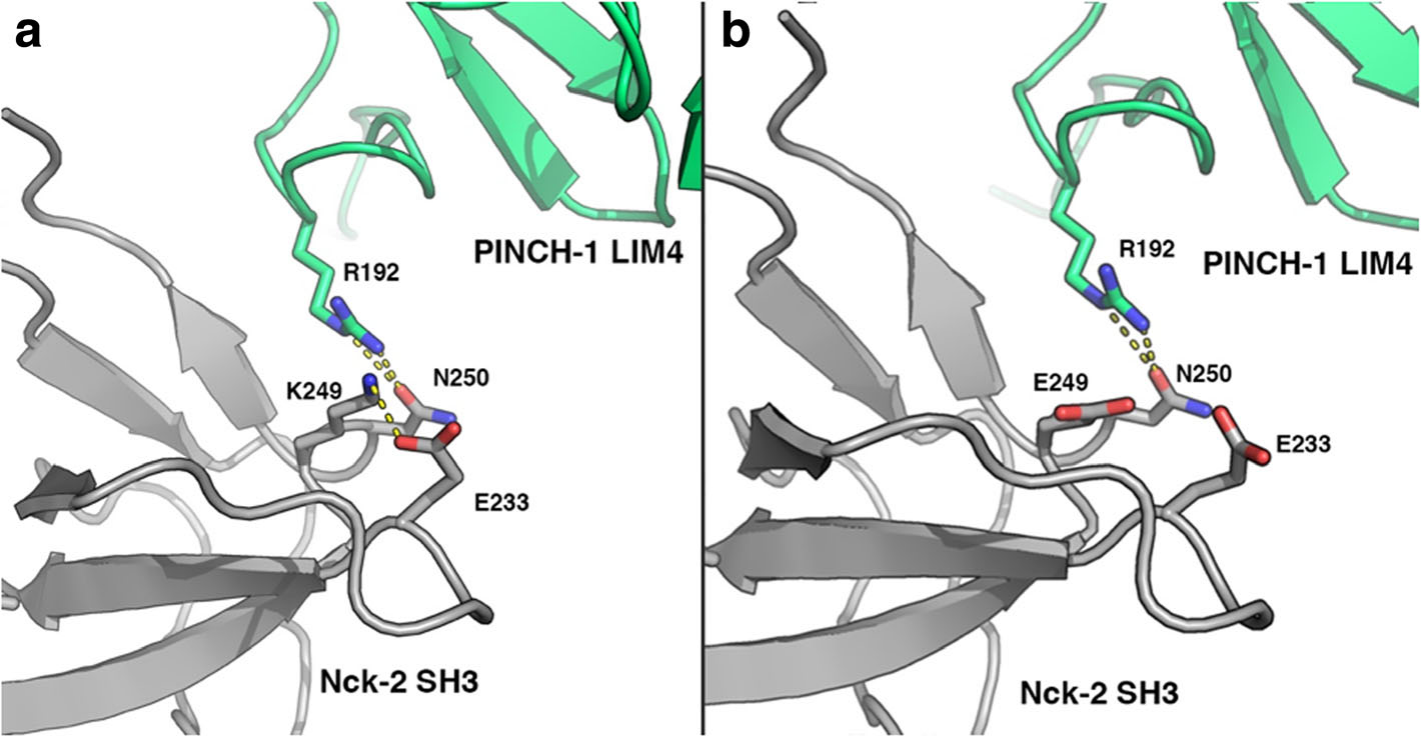
**a** View of the SH3-3/LIM4 binding interface as previously shown by NMR spectroscopy [26]. The Nck2 DH3 domain interface is shown in *gray* and the PINCH-1 LIM4 domain in *green*. K249 interacts with E233 and is part of a network connecting R192 of LIM4 and N250 of SH3. **b** Structural model of the mutant p.K249E and the potential effect in the interactions between SH3 and LIM4

*NCK2* c.745_746delAAinsG has not been annotated in either dbSNP or the 1000 Genomes Project/exome variant server data, and hence, it may be considered to be novel. *NCK2* codes for Human Nck2 (hNck2), a 380-residue adapter protein consisting of three SH3 domains and one SH2 domain. Nck2 plays a pivotal role in connecting and integrating signaling networks constituted by transmembrane receptors, such as ephrinB and effectors critical for cytoskeletal dynamics and remodeling [22–25]. A transient Nck2/PINCH-1 association process has been also reported that may trigger rapid focal adhesion turnover during integrin signaling, mediating cell shape change and migration [26, 27]. We showed that the novel variant identified herein results in a single amino acid change (p.K249E), being this exact amino acid that is reported of fundamental importance during the abovementioned Nck2 SH3 domain protein-protein interactions (Fig. 2a) [22, 26, 27]. p.K249E could severely alter this network of polar interactions and affect the interaction between the two proteins (Fig. 2b). In 2014, Nadalutti et al. [28] observed that celiac patient IgA antibodies disturb the extracellular protein cross-linking function of transglutaminase 2, thus altering cell-extracellular matrix interactions and thereby affecting endothelial cell adhesion, polarization, and motility.

### In silico analyses

To ascertain whether the variants of interest have functional significance, in silico analysis was performed using the SIFT and PROVEAN algorithms [13, 14], as well as Variant Effect Predictor [16] and Regulome DB [8]. As summarized in Table 1, analyses yielded that all variants share an effect to protein structure, which in the case of *NCK2* c.745_746delAAinsG is considered to be damaging. Regulome DB questioned a possible role of the variants of interest in terms of transcription factor binding sites, chromatin states, eQTLs, differentially methylated regions, validated functional SNPs and DNase sensitivity. All variants had minimum or no impact (Additional file 2: Table S1).

**Table 1:** In silico analyses outcome of the six variants of prime interest identified in the family trio

HUGO gene symbol	HGVS description of variant	AA position	Reference residue	Alternative residue	PROVEAN prediction			SIFT prediction		Variant effect predictor			
					Variation type	Score	Prediction	Score	Prediction	Consequence	Impact	PolyPhen	LoFtool
*SLC9A4*	c.1919G>A	640	R	K	Single AA change	−0.67	Neutral	0.140	Tolerated	Frameshift variant	High	–	0.911
*NCK2*	c.745_746delAAinsG	249	K	E	Single AA change	−1.97	Neutral	0.010	Damaging	Frameshift variant	High	–	0.223
*HOXD12*	c.418G>A	140	A	T	Single AA change	−0.01	Neutral	0.201	Tolerated	Frameshift variant	High	–	0.432
*KIAA1109*	c.2933T>C	978	I	T	Single AA change	−0.25	Neutral	0.187	Tolerated	Frameshift variant	High	–	0.845
	c.4268_4269delCCinsTA	1423	T	I	Single AA change	−1.22	Neutral	0.108	Tolerated	Missense variant	Moderate	Benign (0.031)	0.845
*HOXB6*	c.668C>A	223	A	D	Single AA change	−.029	Neutral	0.230	Tolerated	Frameshift variant	High	–	–

Source: PROVEAN v1.1.3 (PROVEAN human genome variants tool, http://provean.jcvi.org/genome_submit_2.php?species=human) and Variant Effect Predictor (http://grch37.ensembl.org/Homo_sapiens/Tools/VEP). Assembly: GRCh37. PROVEAN introduces a delta alignment score based on the reference and variant versions of a protein query sequence with respect to sequence homologs (NCBI NR protein database through BLAST, http://www.ncbi.nlm.nih.gov/). The default score threshold was set at −2.5 for binary classification (deleterious <−2.5 vs. neutral > −2.5). Similarly, (through PSI-BLAST, http://blast.ncbi.nlm.nih.gov/Blast.cgi?CMD=Web&PAGE=Proteins&PROGRAM=blastp&RUN_PSIBLAST=on), SIFT (http://sift.jcvi.org/) may be applied to naturally occurring non-synonymous variants. SIFT score ranges from 0 to 1. A SIFT score of ≤0.05 corresponds to a “damaging” prediction, whereas a SIFT score >0.05 predicts that the variant is likely to be “tolerated.” VEP; Consequence—consequence type of this variation, Impact—a subjective classification of the severity of the variant consequence (high: the variant is assumed to have disruptive impact in the protein, probably causing protein truncation or loss of function or triggering nonsense mediated decay, moderate: a non-disruptive variant that might change protein effectiveness, low: assumed to be mostly harmless or unlikely to change protein behavior, modifier: usually non-coding variants affecting non-coding genes, where predictions are difficult or there is no evidence of impact), PolyPhen—the PolyPhen prediction and/or score, LoFtool—provides a per-gene rank of genic intolerance and consequent susceptibility to disease based on the ratio of loss of function (LoF) to synonymous mutations in ExAC data

### Replication analyses

Replication analyses were carried out in two cohorts to account for population differences. We found that *NCK2* c.745_746delAAinsG is a novel variant that is more abundant in healthy individuals, reaching statistical significance (*P* = 0.03) in the Greek population. Moreover, *HoxD12* c.418G>A, a frameshift variant, was more abundant in pediatric celiac disease patients, reaching statistical significance (*P* < 0.01) in the Serbian population. When celiac disease risk assessment is considered, it is important to note that apart from genotype data, records related to family history along with gender and country of residence should not be disregarded [29]. In both Greek and Serbian patients, *SLC9A4* c.1919G>A and *KIAA1109* c.2933T>C and c.4268_4269delCCinsTA were more abundant in patients; nevertheless, they failed to show statistical significance, possibly due to small sample sizes. All the studied variants satisfied the Hardy-Weinberg equilibrium. All variants were verified as non-frequent ones in agreement with PROVEAN’s scoring scheme, separating disease-associated variants from common ones [14]. Genotype frequencies (%) are summarized in Fig. 1 and Table 2.

**Table 2:** Genotyping data of celiac disease pediatric patients of Greek and Serbian descent and healthy individuals

Population groups	Genotype frequency (%)											
	*HoxB6* c.668C>A			*SLC9A4* c.1919G>A			*KIAA1109* c.2933T>C			*KIAA1109* c.4268_4269delCCinsTA		
	TT	GG	GT	AA	GG	AG	TT	CC	CT	CC/CC	TA/TA	CC/TA
Healthy individuals of Greek descent	0	100	0	0	96	4	86	1	13	100	0	0
Patients of Greek descent	0	100	0	0	93	7	81	2	16	98	0	2
Healthy individuals of Serbian descent	0	100	0	0	97	3	80	0	20	100	0	0
Patients of Serbian descent	0	100	0	0	93	7	81	0	19	100	0	0

In both Greek and Serbian patients, *SLC9A4* c.1919G>A and *KIAA1109* c.2933T>C and c.4268_4269delCCinsTA were more abundant in patients; nevertheless, they failed to show statistical significance, possibly due to a small sample size

## Conclusions

In relation to disease diagnosis and prognosis, data interpretation requires an understanding of the variation in risk-associated variants. In celiac disease, in particular, this knowledge is still largely lacking. As whole-genome and/or whole-exome sequencing approaches begin to be employed in clinical care, the understanding of detected sequence variations on diagnosis (and prognosis) is still not a trivial undertaking. We envisage that the clinical implementation of next-generation sequencing will play a crucial role in delineating inter-individual variability and identification of novel variants towards improved therapeutic modalities. Herein, we propose a multi-step next-generation sequencing-based family genomics approach, similar to our previous conducted cancer genomics study [30], but piloted towards a complex genetic disease, such as celiac disease, to analyze a family trio of Greek descent to identify novel genomic variants of functional significance with the aim of understanding complex disease pathobiology. Recently, we have outlined the paradigm of pharmacometabolomics-aided pharmacogenomics in autoimmune diseases to address the interplay of genomic and environmental influences with information technologies to facilitate data analysis as well as sense- and decision-making on the basis of synergy between artificial and human intelligence. We propose that better-informed, rapid, and cost-effective “omics” studies need the implementation of holistic and multidisciplinary approaches [31].

## Additional files

Additional file 1: A seven-member Greek family has been recruited (informed consents have been obtained), and a trio analysis (III-1, III-2, IV-3) has been performed using the celiac disease model. (PNG 136 kb)
Additional file 2: Regulome DB questioned a possible role of the variants of interest in terms of transcription factor binding sites, chromatin states, eQTLs, differentially methylated regions, validated functional SNPs and DNase sensitivity. All variants had minimum or no impact. (XLSX 3.62 mb)
