# Supplementary figures and images for: Novel genetic risk variants for pediatric celiac disease
Source: Hum Genomics. 2016 Oct 24;10:34. doi: 10.1186/s40246-016-0091-1 (PMC5105295; doi:10.1186/s40246-016-0091-1)

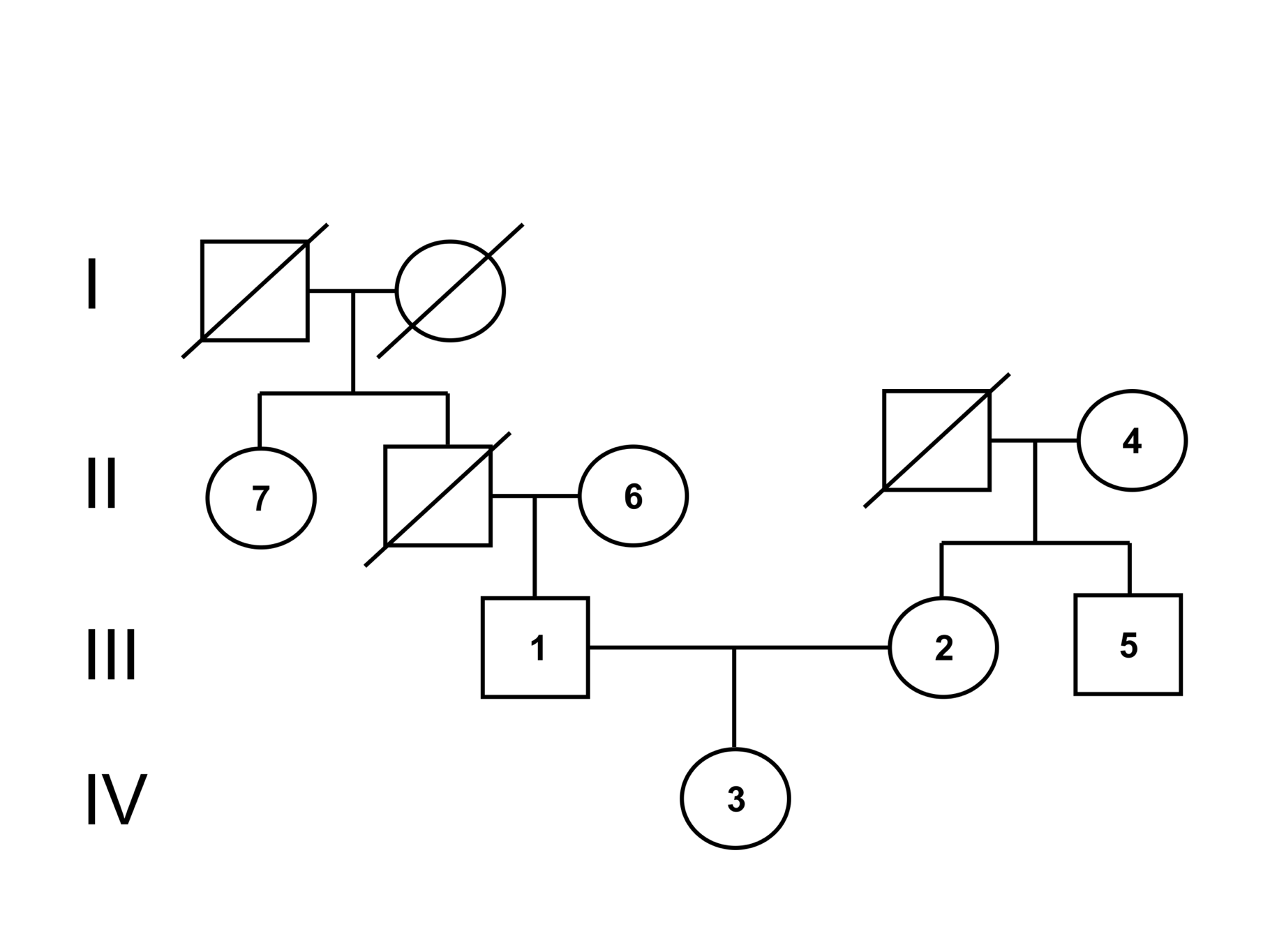

Supplement: Additional file 1: — A seven-member Greek family has been recruited (informed consents have been obtained), and a trio analysis (III-1, III-2, IV-3) has been performed using the celiac disease model. (PNG 136 kb) [file 40246_2016_91_MOESM1_ESM.png]
